# MITOCHONDRIAL DYSFUNCTION IN DYSREGULATED MONOCYTE IMMUNE RESPONSE

**DOI:** 10.1093/geroni/igae098.3674

**Published:** 2024-12-31

**Authors:** Rafael Moura Maurmann, Brandt D Pence

**Affiliations:** University of Memphis, Memphis, Tennessee, United States; University of Memphis, Memphis, Tennessee, United States

## Abstract

Monocytes are key innate immune cells involved in inflammatory signaling. This function is dysregulated in older adults, which thus correlates with increased basal inflammation and disease severity. This phenotype coincides with the buildup of mitochondrial dysfunction markers and defects on mitochondrial dynamics. However, a link between those phenomena remains to be explored. We thus aimed to identify a connection between impaired monocyte immune function and the accumulation of defective mitochondria. We used U937 human monocytic cell line to establish a mitochondrial dysfunction model in vitro comparable to aged monocytes. Cells were treated for 3h with 1 uM FCCP, a mitochondrial depolarized inducer, in the presence or absence of 100 nM bafilomycin A1, an autophagy inhibitor, followed by a 3h recovery in the absence of FCCP. Bafilomycin A1 was kept in cell culture media during recovery. A mitochondrial stress test was then performed on a Seahorse XFp to evaluate extracellular acidification rate (ECAR) and oxygen consumption rate (OCR) responses. FCCP solely treatment led to compromised mitochondrial maximal respiration, which was restored after 3h recovery. However, bafilomycin A1 addition prevented restoration and further aggravated mitochondrial function. The combined treatment induced a decrease in mitochondrial oxygen consumption rate both at basal and stressed conditions, negatively impacting ATP production. Besides, a reduction mitochondrial proton leak may indicate a deficiency on membrane potential maintenance, which further impacts ATP production. While we have not assessed immune function markers to the present, these results validate our model of mitochondrial dysfunction in vitro similar to aged cells.

